# Institutional deliveries in India’s nine low performing states: levels, determinants and accessibility

**DOI:** 10.1080/16549716.2021.2001145

**Published:** 2021-12-16

**Authors:** Ria Saha, Pintu Paul

**Affiliations:** a Public Health Consultant, London, UK; b Centre for the Study of Regional Development, School of Social Sciences, Jawaharlal Nehru University, New Delhi, India

**Keywords:** Institutional delivery, low performing states (LPS), maternal mortality, JSY scheme, NFHS-4

## Abstract

**Background:**

Despite the implementation of several national-level interventions, institutional delivery coverage remains unsatisfactory in India’s low performing states (LPS), leading to a high burden of maternal mortality.

**Objective:**

This study investigates the levels, differentials, and determinants of institutional deliveries in LPS of India. The study also delineates a holistic understanding of barriers to delivery at health facilities and the utilization of the *Janani Suraksha Yojana* (JSY) specifically designed to improve maternal and child health of disadvantaged communities.

**Methods:**

A cross-sectional study was conducted using data from the National Family Health Survey (NFHS)-4, 2015–16. The study was carried out over India’s nine LPS utilizing 112,518 women who had a living child in the past five years preceding the survey. Bivariate and multivariate regression analysis techniques were used to yield findings.

**Results:**

Of the study sample, nearly three-quarters (74%) of women delivered in a health institution in the study area, with the majority delivered in public health facilities. The multivariate analysis indicates that women who lived in rural areas, belonged to disadvantaged social groups (e.g. Scheduled caste/tribes and Muslims), and those who married early (before 18 years) were less likely to utilize institutional delivery services. On the other hand, women’s education, household wealth, and exposure to mass media were found to be strong facilitators of delivering in a health facility. Meeting with a community health worker (CHW) during pregnancy emerged as an important predictor of institutional delivery in our study. Further, interaction analysis shows that women who reported the distance was a ‘big problem’ in accessing medical care had significantly lower odds of delivering at a health facility.

**Conclusions:**

The study suggests emphasizing the quality of in-facility maternal care and awareness about the importance of reproductive health. Furthermore, strengthening sub-national policies specifically in underperforming states is imperative to improve institutional delivery coverage.

## Background

Maternal mortality remains a major public health problem and a challenging concern in low- and middle-income countries (LMICs) [1]. Pregnancy and childbirth-related complications are the leading causes of maternal mortality [2–4]. Majority of these preventable maternal deaths (about 94%) occur in resource-constrained settings of LMICs [1]. Although India has made considerable progress in reducing maternal mortality over the past years (from 556 deaths per 100,000 live births in 1990 to 113 deaths per 100,000 live births in 2018), it remains alarmingly high in low performing states (LPS) (161 deaths per 100,000 live births) [5–8]. The LPS in India include Assam and eight Empowered Action Group (EAG) states (i.e. Bihar, Chhattisgarh, Jharkhand, Madhya Pradesh, Odisha, Rajasthan, Uttar Pradesh, and Uttarakhand).

Despite several national and state-specific policy initiatives and implementation measures, disparity prevails across sub-national regions regarding utilization of essential maternal healthcare services resulting in failure to achieve the *National Health Policy 2017* target of reducing maternal mortality ratio (MMR) to 100 deaths per 100,000 live births [5,8–10]. Safe delivery care (Institutional delivery/delivery by skilled health attendant [SBA]) is an important component of the continuum of maternal healthcare that averts preventable maternal and neonatal deaths with adequate health-seeking and good quality of care [11]. Although India’s achievements toward utilization of institutional delivery services have been substantial over the years (increased from 41% in 2005–06 to 79% in 2015–16) with large-scale public health investments, studies suggest that usage of facility-based skilled delivery care is uneven across states, socio-economic strata, and rural-urban residence [5,6,12–15]. A large amount of expenditure incurred at the point of healthcare use through high out-of-pocket payments (OOPs) and associated catastrophic health expenditure (CAH) (increased from 11% in 1995 to 25% in 2014) hinder beneficiaries (especially from marginalized communities) from delivering at health facilities and forcing them to uptake unsafe home deliveries [6,16,17]. Place of delivery has been observed to be significantly associated with maternal and child health outcomes where home deliveries (in absence of SBA) increase the likelihood of delivery complications with adverse maternal and perinatal outcomes [18–20]. As per the recent National Family Health Survey (NFHS-4, 2015–16), home deliveries account for 26% and 21% of all childbirths in LPS and India respectively, which potentially increase the risk of preventable maternal and neonatal deaths, especially in high-burden states such as Bihar, Uttar Pradesh, Madhya Pradesh, Chhattisgarh and Odisha [15].

With the aim of reducing high OOPs/CAH and improving maternal and neonatal health, the Government of India has launched numerous ‘Safe Motherhood’ programmes including the *Janani Suraksha Yojana* (JSY) intervention in 2005 to escalate the demand for nation-wide institutional delivery services and expand its utilization especially among socio-economically disadvantaged women [21]. It is implemented as India’s flagship scheme under National Rural Health Mission [NRHM] to address the socio-economic inequality in the utilization of institutional delivery services through centrally sponsored conditional cash transfer mechanisms targeted to pregnant women delivering at public health facilities and accredited private institutions enrolled under JSY [4,22–24]. The regulations of JSY differ across states – in LPS, all rural/urban women are eligible to utilize the JSY scheme delivering in government health centres or accredited private institutions irrespective of socioeconomic status, age, and parity; but in high performing states, JSY cash assistance is only restricted to women belonging to below poverty line (BPL) and Scheduled caste (SC) and Scheduled tribe (ST). The cash transfer amount is higher in LPS with a financial incentive of $19 (rural) and $13.6 (urban) compared to half the amount in better-off states [18]. Apart from financial rewards upon delivery and throughout postnatal care, the programme also supports beneficiaries with free (emergency) transport facilities to health institutions and integrates Accredited Social Health Activists (ASHAs) into the scheme through prenatal and postnatal care cascade with an additional cash assistance compensation of $8.2 and $2.7 in rural and urban areas respectively in LPS [22,25,26].

Whilst post-JSY scheme implementation, institutional delivery service has been widely utilized in India with extensive monetary assistance from the government, several beneficiaries still find accessing the JSY scheme challenging and unhelpful (sometimes with untimely cash disbursement) [5,23,27,28]. Hence, even a decade later institutional delivery services and utilization of JSY remain unequal and uneven within states and regions where beneficiaries still struggle to access the healthcare facilities due to insufficient logistic support and suffer from discriminatory/unsatisfactory (poor) quality of care at public healthcare facilities [23,29,30]. A study conducted in India’s nine LPS by Randive et al. [26] found no significant association between the rise in institutional deliveries post JSY inception and reduction of district-level MMR. The study findings essentially emphasize the possibility of potentially other context-specific contributing factors (for example, poor quality of care, ill-equipped healthcare facilities among several others) associated with the rise in maternal deaths which is outside the scope of the JSY scheme. Majority of pregnant women (especially from marginalized communities) are frequently exposed to ‘too little, too late’ or disrespectful maternity care which implies an absence of requisite person-centered maternity care as specified by the WHO framework on quality of maternity care in all public health facilities [26,31–36]. Exposure to mistreatment and derogatory services make the process of childbirth a negative and tragic experience for the mothers deterring further institutional deliveries and necessary postpartum visits [31,32]. A recent study by Rao et al. [37] conducted in Uttar Pradesh (India) observed that even though beneficiaries from the poor household value (higher) cash rewards the most, they are willing to sacrifice the size of financial rewards at the cost of high-quality health services received.

Definite supply-side (insufficient transport facilities especially in cases of emergencies) and resource-constrained barriers (absence of/insufficient skilled birth attendants and basic/comprehensive emergency obstetric care functionality) limit the uptake of institutional deliveries and associated JSY scheme resulting in poor maternal health outcomes in specific states and socio-economic groups [20,23,38–40].

Previous studies indicate that resource-constrained facility functioning and associated poor quality of care emerge as strong determinants associated with underuse of nearest primary health centres and bypassing to other tertiary level care for childbirth [20,41–43]. Sabde et al [20] found that although the JSY scheme has substantiated to be a powerful intervention in increasing demand for in-facility births in Madhya Pradesh (82%, NFHS 2015–16), structural and facility-level constraints (unavailability/time inefficiency of free transportation and abysmally functional facility) were significantly associated with chronic underuse of and barriers to nearest primary level health facility-based care. Despite the committed provision of free transportation (Janani Express Programme) under the JSY scheme, several women experienced significant transport-related delays in reaching the health facility with 42% at home and 52% in-transit preventable maternal deaths recorded [20,43,44].

Overall, previous studies essentially reinforce that JSY is not one solution for all towards improvement in institutional deliveries (and reduction in MMR) since patterns of service usage are integrally associated with structural, technical, and resource-constrained factors which outweigh JSY’s scope of involvement and individual-level determinants. Findings of early studies have provided an understanding of the nationwide determinants/factors of institutional deliveries and highlighted a comparatively low utilization of services and disproportionately higher MMR in LPS. Hitherto, to the best of our knowledge, no study attempted to analyse the specific determinants (including intermediate factors) and associated barriers to persistent low utilization of institutional deliveries across LPS in the context of post-JSY. Therefore, this paper aims to investigate levels, differentials, and determinants (socio-demographic characteristics and intermediate factors) of (low) institutional deliveries in LPS using a large-scale population-based survey (NFHS-4, 2015–16). The study also demonstrates a holistic understanding of the predominant resource-constrained and technical barriers to deliver at healthcare facilities utilizing the JSY scheme which will essentially inform policy framework development pathways towards effective equitable use of services in this particular region.

## Methods

### Data source

We used data from the fourth round of India’s Demographic and Health Survey (DHS), known as the National Family Health Survey (NFHS)-4, conducted from January 2015 to December 2016. The NFHS-4 is a large-scale, nationally representative sample survey covering all 29 states and 7 union territories of India. It was carried out by the International Institute for Population Sciences (IIPS), Mumbai, under the stewardship of the Ministry of Health and Family Welfare (MoHFW), Government of India. The survey provides essential information on population, health, and family welfare, such as household characteristics, fertility and fertility preferences, utilization of maternal healthcare services, maternal mortality, nutrition and anaemia, family planning methods, child health status, non-communicable diseases, women’s autonomy, and domestic violence [15]. For the present study, we utilized data on place of delivery, financial assistance during delivery (e.g. JSY), reasons for not delivering in a health facility, and background information of the respondents.

In addition, we used data on state-level maternal mortality ratio (MMR), drawn from the bulletin on maternal mortality in India (2016–2018) of the Sample Registration System (SRS), Office of the Registrar General, India [7].

### Sampling design

In NFHS-4, a two-stage stratified sampling design was adopted for the selection of the participants. In total, 28,586 clusters (primary sampling units) were chosen, of which fieldwork was done for 28,522 clusters. The 2011 Census enumeration served as the sampling frame for the selection of clusters. In the first stage, the clusters were selected using probability proportional to size (PPS). In the second stage, a complete household mapping and the listing was prepared in the selected clusters, and 22 households were randomly chosen in each cluster from the household listing. A detailed description of the sampling design and survey procedure is provided in the NFHS-4 national report [15].

### Study setting

**Figure 1.**
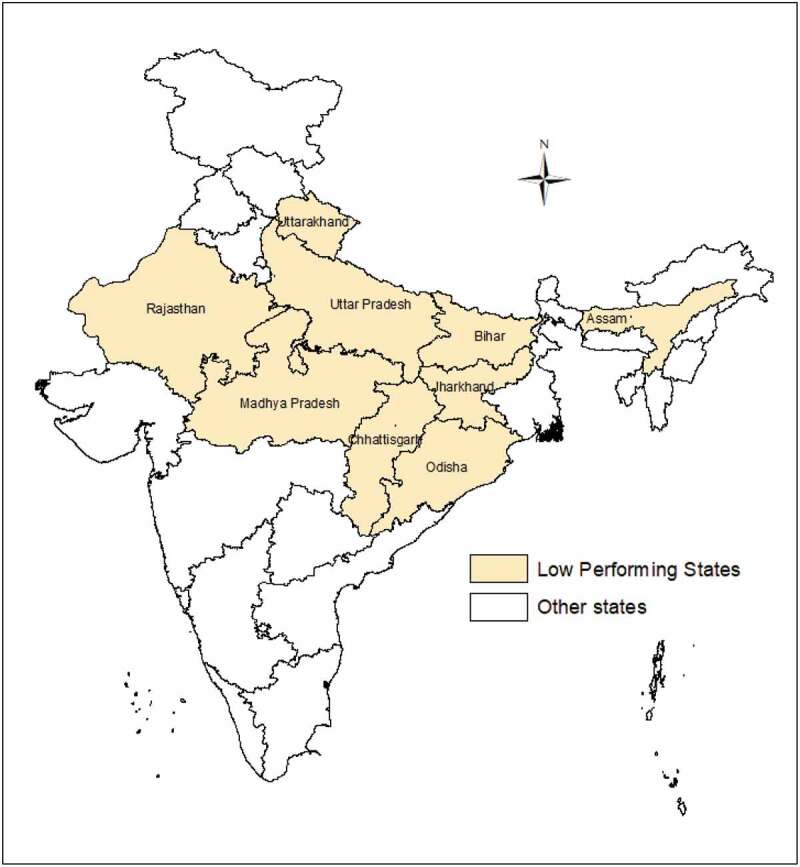
Map showing geographical location of the study area (LPS) in the country

The present study was conducted in India’s nine under-performing states in maternal and child health outcomes which are also called LPS. The *beige*-coloured regions marked in the map show geographical location of the study region (Figure 1). These states constitute about half of the country’s population [43]. Among these states, Uttar Pradesh is the most populous state (200 million) followed by Bihar (104 million) and Madhya Pradesh (73 million); Uttarakhand has the lowest population (10 million) [45]. A large segment of the population (11–40%) lives below the poverty line in these states [46]. More than three-quarters of mothers did not get full antenatal care in any of these nine states in 2015–16 [15]. The LPS region accounts for 12% of global maternal deaths [26]. According to India’s Annual Health Survey (AHS), these states account for about 62% of maternal deaths, 71% of infant deaths, 72% of under-five deaths, and 61% of births in the country [45]. These nine states have been entitled as LPS for focused attention in order to reduce the high burden of maternal and child deaths by promoting institutional delivery services. Therefore, these specific states were chosen in the present study to understand the determinants of and barriers to persistent low institutional delivery coverage in order to suggest some data-informed policy interventions to reduce the burden of maternal mortality in the study setting.

### Study sample

**Figure 2.**
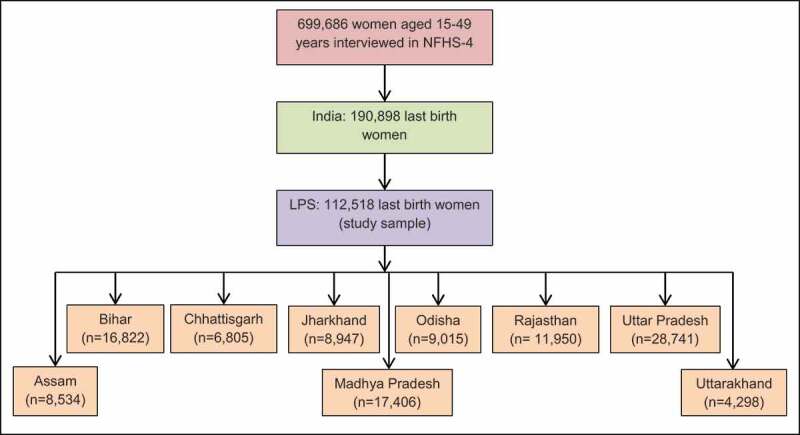
Selection of study participants, NFHS-4 2015–16

The NFHS-4 interviewed 699,686 women aged 15–49, with a response rate of 97% in 601,509 households. For the whole country, data on the utilization of maternal health care including delivery care were collected from 190,898 women aged 15–49 years who had a living child in the past five years preceding the survey. Since our study focused on nine LPS, we limited our sample to 112,518 last birth women (age 15–49) in the past five years. Among nine LPS, Uttar Pradesh (n = 28,741) comprised the largest share of the sample followed by Madhya Pradesh (n = 17,406), while Uttarakhand (n = 4,298) represented the lowest number of participants. Figure 2 illustrates the selection of study participants.

### Variable(s) selection

**Figure 3.**
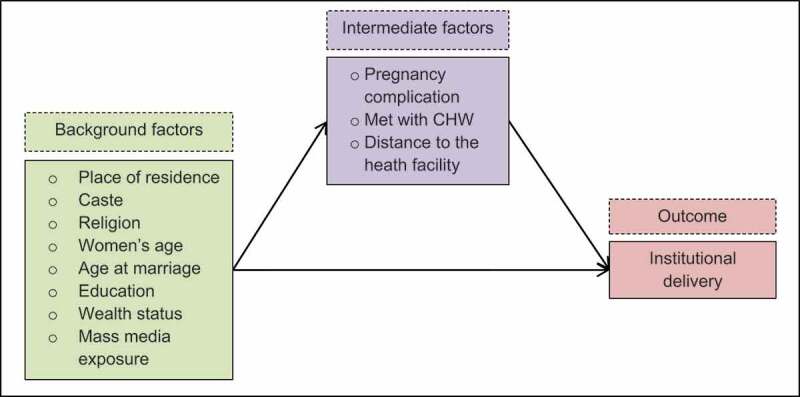
Conceptual framework showing the influence of background and intermediate factors on institutional delivery

We conducted a comprehensive literature search in databases like PubMed, Google Scholar, and Global Health databases to identify and understand the determinants/factors influencing institutional deliveries across nine LPS of India. After comprehending the various factors from an in-depth literature review, we classified the factors influencing institutional deliveries in India into background and intermediate factors through the construction of a conceptual framework (Figure 3).

#### Outcome variable(s)

Institutional delivery was the outcome variable in this study. In NFHS-4, women were asked about their place of delivery. Place of delivery was categorized as home, public health sector, private health sector, NGO/trust hospital, and others. The public health sector includes the Govt./municipality hospital, Govt. dispensaries, UHC/UHP/UFWC, CHC/rural hospital, block PHC, sub-centre, and other public health facilities. The private health sector includes private hospitals, maternity homes, clinics, and other private sector health facilities. Women who gave birth to any public or private health sector or NGO/trust hospital/clinic were considered as delivered in a health institution. Women who delivered in a health facility were coded as ‘1ʹ and those who gave birth at home and ‘others’ (other than a health facility) were coded as ‘0ʹ.

#### Independent variable(s)

To assess determining factors associated with institutional delivery, we have incorporated several socio-demographic characteristics and intermediate predictor variables in this study. Socio-demographic variables include place of residence, caste, religion, women’s age, age at marriage, educational level of women, household wealth status, and exposure to mass media. Place of residence was categorized as urban and rural. Respondents’ caste status was divided into four broad groups: SC, ST, Other Backward Class (OBC), and other (forward caste). Religious affiliation of women was categorized as Hindu, Muslim, and other religions. Women’s age was grouped into 15–24, 25–34, and 35–49 years. Age at marriage was classified into two groups: below 18 (child marriage) and 18 and above (adulthood marriage). Women’s educational attainment was categorized into four levels of education: no education (illiterate), primary level (1–5 grades), secondary level (6–12 grades), and higher education (13+ grades). Wealth quintile/index is a measure of a household’s standard of living. The NFHS-4 assessed the wealth index from the ownership of consumer items including dwelling characteristics and accessibility to services. A score has been assigned to each individual using principal component analysis. Based on these wealth scores, participants have been classified into five quintiles; each represents 20% of the respondents. These five wealth quintiles from bottom to top are poorest, poorer, middle, richer, and richest. Women’s exposure to mass media was assessed from the frequency of reading newspapers/magazines, listening to radio, and watching television. Based on access to these three media, women were categorized into three groups: no exposure (none of the media accessed), partial exposure (one or two media accessed), and full exposure (all three media accessed).

Further, we have included three important intermediate predictor variables in the analysis. These are pregnancy complications, met with any community health worker (CHW), and perceived distance to the health facility.

We derived women’s experience of pregnancy complications during the last pregnancy from the following three sets of questions: (1) *‘Did you have difficulty with your vision during daylight?’* (2) *‘Did you have convulsions, not from fever?’* (3) *Did you have swelling of the legs or body?”* A dichotomous variable (yes/no) was constructed where women who reported any of these complications were coded as ‘1ʹ (experienced any pregnancy complication) and otherwise coded as ‘0ʹ (not experienced any complication). With regard to meeting with CHW, women were enquired whether they met with any CHW during the last three months of pregnancy, and it was dichotomized into yes (coded as ‘1ʹ) or no (coded as ‘0ʹ). CHWs include Auxiliary nurse-midwife, lady health visitor, ASHA, Anganwadi worker, or other community health workers. To understand the question of accessibility to medical care, women were asked to what extent they face difficulty regarding the distance to the health facility when they are sick and want to get medical advice or treatment. The responses of participants were recorded as follows: a big problem, a small problem, and no problem.

### Statistical analysis

We performed descriptive statistics to show the frequency and percentage distribution of study participants by selected explanatory variables. To assess the coverage and differentials in institutional deliveries, we estimated institutional delivery rate (%) by socio-demographic characteristics and intermediate factors for all nine states, and the differences were measured by Pearson’s chi-square statistics. The percentage distribution of institutional deliveries by the public-private sector has also been estimated across LPS. Multivariate logistic regression models were employed to examine the influence of socio-demographic characteristics and intermediate factors for each focused state. We also included the ‘state variable’ in the multivariate regression model (analysis which included all nine states) to control the subnational (state-level) variations in the analysis. To further understand the structural barriers to access a health institution for institutional delivery, we performed additional analyses. First, we estimated self-reported reasons for not delivering in a health facility across all nine LPS. Second, women’s perceived distance to the health facility was analysed to evaluate their accessibility for medical care. Interaction models (bivariate and multivariate) were employed to assess whether the place of residence and distance to the health facility plays a critical role in determining institutional delivery utilization in LPS. In addition, the distribution, and differentials of JSY were assessed since it is an important Safe Motherhood policy initiative scheme to improve institutional delivery coverage. The differentials in JSY service utilization by various background characteristics were tested by Pearson’s chi-square test. Finally, the multivariate logistic regression model was performed to assess the likelihood of JSY utilization by socio-demographic characteristics of women. We checked for multicollinearity between the independent variables using variation inflation factors and found no evidence of collinearity problems in the analysis. The results of logistic regression models have been presented in odds ratio (OR) with a 95% confidence interval (CI). All the statistical analyses were performed using the STATA version 14.0 (StataCorp LP, College Station, TX, USA).

## Results

### Descriptive statistics

Table 1 presents the sample distribution of the study participants. In the present study, majority of the participants were living in rural areas (81%) and Hindu (82%). Just over half of them belonged to the OBC category (51%). About one-third of women (33%) were in the younger age group (15–24 years) and more than one-half (56%) were in the age group of 25–34 years. About two-fifths of the study participants (40%) had no formal education, while only 9% had a higher level of education. More than half of the women (54%) got married before attaining the legal marriage age (below 18 years). Most of them belonged to the middle to poorest (76%) wealth indices. About 38% of women had no mass media exposure and over half of the women (53%) met with at least one CHW during the last three months of pregnancy. More than half of the pregnant women (54%) did not face any pregnancy complications and about 38% of pregnant women felt that accessing a health institution for seeking treatment was a ‘big problem’.

**Table 1.** Descriptive statistics for the study participants, LPS, NFHS-4 2015–16 (n = 112,518)

Characteristics	N (Sample)	% (Percentage)
**Place of residence**		
Urban	22,784	19.3
Rural	89,734	80.8
**Caste**		
SC	21,748	21.1
ST	17,079	11.6
OBC	51,817	50.5
Other	18,616	16.8
**Religion**		
Hindu	92,775	82.5
Muslim	16,941	15.8
Other	2,802	1.6
**Age (years)**		
15–24	38,065	33.1
25–34	62,181	55.5
35–49	12,272	11.4
**Age at marriage (years)**		
Below 18	49,044	45.7
18 and above	61,834	54.3
**Education**		
No education	41,996	39.7
Primary	16,910	14.7
Secondary	43,954	36.9
Higher	9,658	8.7
**Wealth index**		
Poorest	39,165	36.5
Poorer	27,730	24.3
Middle	19,061	16.4
Richer	14,426	12.5
Richest	12,136	10.4
**Mass media exposure**		
No	39,624	37.7
Partial	66,930	56.7
Full	5,964	5.6
**Pregnancy complication**		
No	62,798	54.8
Yes	49,525	45.2
**Met with CHW**		
No	56,314	53.4
Yes	56,167	46.6
**Distance to health facility**		
No problem	31,370	27.5
Small problem	39,249	34.6
Big problem	41,899	37.9

Note: Numbers are un-weighted and percentages are weighted.

### Maternal mortality in LPS and other states

**Figure 4.**
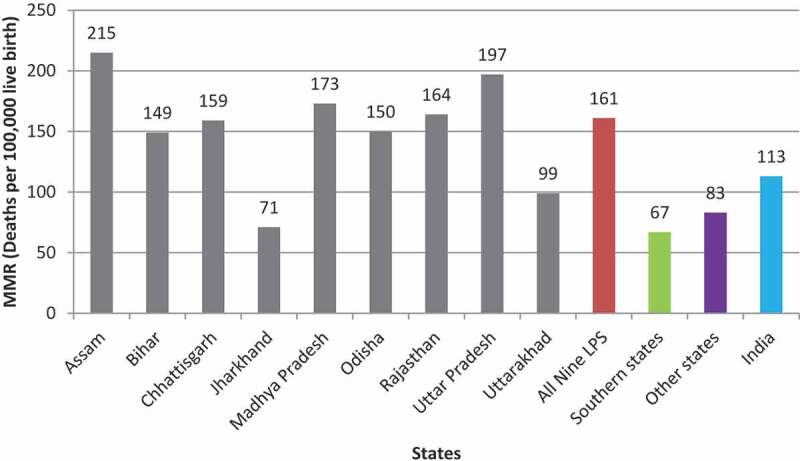
Maternal mortality ratio (MMR) in LPS and other India states, SRS 2016–18

The MMR in India’s nine LPS is substantially higher (161) than the national average (113), southern states (67), and other states (83). Among all LPS, Assam represented the highest MMR (215) followed by Uttar Pradesh (197) and Madhya Pradesh (173), while Jharkhand (71) and Uttarakhand (99) had comparatively lower rates of maternal deaths (Figure 4).

### Institutional delivery coverage and public-private gap

**Figure 5.**
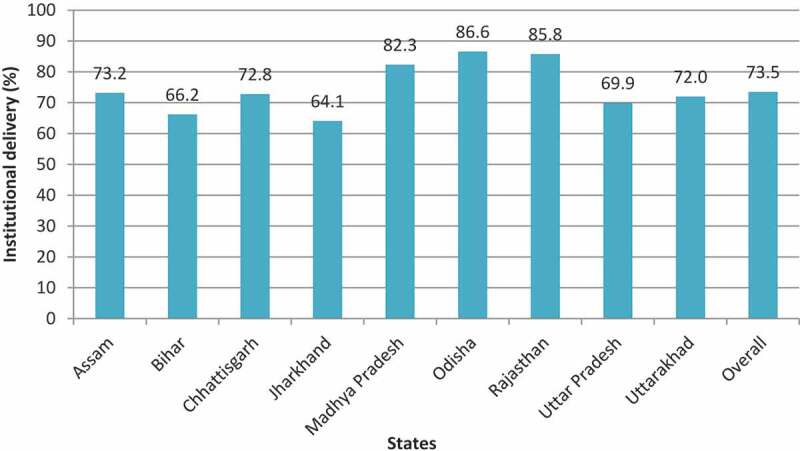
Coverage of institutional delivery (%) across LPS, NFHS-4 2015–16

**Figure 6.**
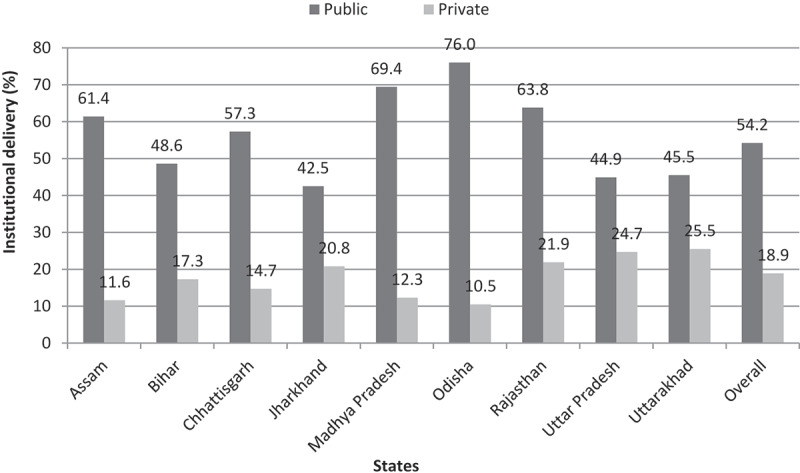
Distribution of institutional delivery (%) by public-private sector across LPS, NFHS-4 2015–16

Figure 5 shows institutional delivery coverage (% distribution) in focused states and is highest in the state of Odisha (87%) followed by Rajasthan (86%) and Madhya Pradesh (82%). States like Assam (73%), Chhattisgarh (73%), Uttarakhand (72%), and Uttar Pradesh (70%) are average performing states with Bihar (66%) and Jharkhand (64%) being comparatively low performers. Figure 6 shows a striking gap between the public and the private sector in institutional delivery. Women majorly utilized public health facilities in Odisha (76%) followed by Madhya Pradesh (69%) and Rajasthan (63%). The share of institutional delivery in the private sector was highest in Uttarakhand (26%) followed by Uttar Pradesh (25%), and Rajasthan (22%).

### Differentials in institutional delivery coverage

Table 2 depicts the estimated institutional delivery rate (%) by socio-demographic characteristics and intermediate factors across nine LPS. The utilization of institutional deliveries seems to be lowest in the rural areas of Jharkhand (59%) followed by Bihar (65%) and Chhattisgarh (69%). Overall, the percentage of institutional delivery was found to be comparatively low among older women (35–49 years) (60%), while the percentage was high among the younger aged women (15–24 years) (79%). Women who were Muslim (62%) and from SC (66%) were less likely to deliver at an institution than women who were Hindu (76%) and from other castes (80%). The institutional delivery rate was high among the women who were married at 18 years and above (78%) and have a higher education background (94%). Even with different government initiatives like JSY and various other (government aided) maternity benefit programmes, the institutional delivery services are very poorly utilized by certain sectors of women in all the nine LPS especially those who are at the lowest wealth quintile (60%) and with lack of any formal education background (60%). Numerous systematic factors most importantly lack of health awareness and insufficient accessibility to antenatal care services may have resulted in this. Exposure to mass media seems to play an important role in health awareness aiding access to institutional delivery services as women who were completely exposed to mass media utilized the services the most in all the focused states (87%) than those who had no exposure at all (61%). Intermediate factors like pregnancy complications do not play a significant role here, but women who underwent pregnancy complications were slightly more likely to deliver at a healthcare facility (75%) than those who did not (73%). Similarly, women who had an interaction with CHW in any of the antenatal care sessions or home visits were more likely to deliver at a health facility (79%) than other women (69%). Challenges to access health facilities like a long distance to the health facility hinder women to utilize the life-saving services as women were less likely to deliver at an institution who thought the distance was a ‘big problem’ for them (67%) than women for whom it was ‘a small problem’ (75%) or ‘no problem’ at all (81%).

**Table 2.** Percentage distribution of institutional deliveries by selected characteristics across LPS, NFHS-4 2015–16

Characteristics	Assam (n = 8,534)	Bihar (n = 16,822)	Chhattisgarh (n = 6,805)	Jharkhand (n = 8,947)	Madhya Pradesh (n = 17,406)	Odisha (n = 9,015)	Rajasthan (n = 11,950)	Uttar Pradesh (n = 28,741)	Uttarakhand (n = 4,298)	All nine LPS (n = 112,518)
**Place of residence**										
Urban	93.4	77.2	84.8	83.6	94.5	90.1	91.7	74.2	81.5	83.1
Rural	70.7	64.9	69.4	59.1	77.7	86.0	84.0	68.8	67.2	71.2
**Caste**										
SC	85.0	60.1	71.4	59.3	85.2	87.2	86.5	66.7	65.9	71.0
ST	81.1	56.6	64.9	50.9	61.6	74.8	78.5	51.6	72.9	65.6
OBC	85.4	67.7	76.3	69.6	88.4	92.6	85.9	69.1	69.1	74.4
Other	66.0	71.9	90.1	82.0	92.2	92.9	91.3	78.9	76.4	80.3
**Religion**										
Hindu	86.2	68.9	72.2	67.1	81.5	87.5	86.4	71.7	73.1	75.7
Muslim	55.6	53.7	91.0	62.6	90.9	74.5	78.4	62.6	65.0	62.6
Other	71.0	59.0	76.9	49.1	94.2	69.4	97.0	89.6	87.6	67.1
**Age (years)**										
15–24	75.7	72.9	76.5	70.4	84.0	89.4	89.1	76.2	73.4	78.7
25–34	74.3	65.3	71.7	61.7	82.4	86.8	85.4	70.1	73.1	73.2
35–49	61.3	52.2	64.8	50.3	73.2	77.3	73.0	56.7	58.5	59.9
**Age at marriage (years)**										
Below 18	65.5	63.7	63.2	59.5	76.7	81.0	82.7	63.9	60.7	68.2
18 and above	80.7	69.3	78.4	69.0	87.5	89.6	89.2	74.9	76.4	78.4
**Education**										
No education	51.6	57.4	56.9	46.3	69.0	68.3	76.5	57.5	53.7	60.4
Primary	62.5	66.4	67.0	58.7	80.9	85.7	86.9	66.9	59.4	71.9
Secondary	82.5	79.2	78.3	76.8	90.0	94.8	92.7	79.2	74.5	83.5
Higher	97.3	92.5	96.5	92.7	98.2	97.8	97.6	91.2	93.2	93.9
**Wealth index**										
Poorest	52.2	57.9	57.9	50.2	65.5	75.3	72.9	58.4	44.5	60.1
Poorer	73.7	71.3	71.8	70.3	83.6	88.9	82.2	68.7	53.6	74.4
Middle	87.3	79.4	77.8	75.4	91.0	95.5	87.5	72.6	64.8	80.8
Richer	93.5	85.3	83.4	87.1	93.2	97.7	91.2	77.2	76.2	85.3
Richest	98.1	92.3	92.8	95.6	97.9	97.7	96.6	88.4	91.5	92.9
**Mass media exposure**										
No	56.0	60.0	53.7	50.5	66.4	72.0	74.0	60.7	51.8	61.4
Partial	83.0	73.4	75.6	74.3	87.3	90.4	89.7	75.1	73.3	80.2
Full	88.9	80.3	86.2	84.0	93.8	97.3	94.9	85.5	84.5	87.4
**Pregnancy complication**										
No	73.2†	65.5	69.7	63.0	81.3	65.3	84.4	69.4	70.1	72.7
Yes	73.3†	67.1	76.7	65.7	83.6	88.1	88.2	70.5	74.6	74.6
**Met with CHW**										
No	64.3	63.7	68.9	60.4	78.4	84.2	82.9	65.5	69.1	68.8
Yes	78.8	71.6	74.0	68.3	85.9	87.4	89.8	76.1	75.6	79.0
**Distance to health facility**										
No problem	84.8	72.8	79.0	77.8	88.9	91.5	89.9	75.2	79.3	80.5
Small problem	75.1	70.6	75.1	67.8	82.5	88.7	86.3	70.5	73.7	75.4
Big problem	62.8	59.7	63.8	56.6	76.4	81.9	80.3	64.8	62.0	66.7

Note: All percentage differentials are significant at *p* < 0.05 (derived from Pearson’s chi-square test) otherwise indicated by †.

### Factors influencing institutional delivery

Table 3 presents the results of multivariate regression analysis performed to examine the influence of socio-demographic characteristics and intermediate factors on institutional delivery across nine LPS of India. Overall, rural women were less likely to deliver at a healthcare facility (OR: 0.94; 95% CI: 0.89, 0.98) than those from urban areas. Women residing in rural areas of Assam (OR: 0.52, 95% CI: 0.36, 0.75), Madhya Pradesh (OR: 0.58, 95% CI: 0.49, 0.69) and Uttarakhand (OR: 0.66, 0.54, 0.81) had substantially lower odds of institutional delivery, whereas rural women of Uttar Pradesh (OR: 1.31, 95% CI: 1.21, 1.42) were more likely to deliver in a health facility than urban women. Social status of the respondents (i.e. religion and caste) is significantly associated with institutional delivery. Overall, women who were Muslim (OR: 0.57; 95% CI: 0.55, 0.60) or other religions (other than Hindu and Muslim) (OR: 0.86; 95% CI: 0.78, 0.95) had lower odds of institutional delivery than Hindus. Except for Chhattisgarh, Muslim women were significantly less likely to deliver in a health facility for eight other states. Overall, women who belonged to SC (OR: 85, 95% CI: 0.81, 0.90) and ST (OR: 0.58, 95% CI: 0.54, 0.61) were less likely to utilize institutional delivery services than other caste women. However, in Rajasthan, women from SC, ST, and OBC had higher odds of delivering in a health facility than others. The odds of institutional delivery were lower among older women as compared to younger women for all focused states. Women who were married early (<18 years) are less likely to utilize institutional delivery services (OR: 0.78; 95% CI: 0.76, 0.80) than women who were married at 18 years and above and is consistent with all nine states. Women who had higher education backgrounds (OR: 3.77; 95% CI: 3.42, 4.16) and belonged to the richest wealth index (OR: 3.45; 95% CI: 3.15, 3.78) preferred to deliver at an institution more than others. The influence of educational attainment appeared to be strongest in Assam and Chhattisgarh, where women with a higher level of education were about five times more likely to deliver in a health facility than women who had no education. In Assam, women from the richest wealth index were almost 14 times more likely to deliver in a health institution than those from the poorest wealth index. Similarly, the odds of delivering in a health facility among the richest women were about five to six-fold higher in states likes Jharkhand, Madhya Pradesh, and Uttarakhand than the poorest women. Overall, exposure to mass media had a positive relationship with institutional delivery utilization. However, state-specific findings showed a weak relationship between women’s exposure to mass media and institutional delivery services, particularly for Assam, Bihar, Chhattisgarh, Jharkhand, and Uttarakhand. Intermediate factors (i.e. pregnancy complications, meeting with any CHW, and distance to the health facility) are also significantly associated with institutional delivery services. Women who experienced any pregnancy complication (OR: 1.13; 95% CI: 1.10, 1.17) and those who met with any CHW (OR: 1.63; 95% CI: 1.58, 1.68) preferred to deliver more in a health facility. Although pregnancy complication was not significant for Assam and Jharkhand, meeting with a CHW consistently had a positive relationship with the utilization of institutional delivery services for all focused states. Distance to the health facility also significantly influences the likelihood of utilizing institutional delivery services where women who reported distance to a health facility was a ‘big problem’ did not prefer to deliver at a health institution (OR: 0.79; 95% CI: 0.76, 0.82) than those who perceived distance to a health facility was ‘not a problem’. Women who felt that the distance was a ‘big problem’ in assessing a health facility consistently had a lower likelihood of institutional delivery services for all nine states. Additionally, state-level variations of institutional delivery exhibited that compared to women from Uttarakhand, women who resided in other eight states were two to five times more likely to deliver in a health facility.

**Table 3.** Multivariate logistic regression models assessing socio-demographic and intermediate factors influencing institutional deliveries across LPS, NFHS-4 2015–16

Variables	Assam OR (95% CI)	Bihar OR (95% CI)	Chhattisgarh OR (95% CI)	Jharkhand OR (95% CI)	Madhya Pradesh OR (95% CI)	Odisha OR (95% CI)	Rajasthan OR (95% CI)	Uttar Pradesh OR (95% CI)	Uttarakhand OR (95% CI)	Overall OR (95% CI)
***Socio-demographic factors***										
**Place of residence**										
Urban (Ref.)										
Rural	0.52 (0.36–0.75)**	0.92 (0.81–1.06)	0.99 (0.83–1.18)	0.72 (0.61–0.85)**	0.58 (0.49–0.69)**	0.99 (0.77–1.28)	0.97 (0.82–1.16)	1.31 (1.21–1.42)**	0.66 (0.54–0.81)**	0.94 (0.89–0.98)**
**Caste**										
SC	0.83 (0.62–1.12)	0.61 (0.53–0.69)**	0.53 (0.36–0.78)**	0.58 (0.45–0.75)**	1.03 (0.84–1.27)	0.70 (0.50–0.97)*	1.28 (1.04–1.57)*	0.73 (0.67–0.80)**	0.83 (0.69–0.99)*	0.85 (0.81–0.90)**
ST	0.72 (0.55–0.93)*	0.56 (0.45–0.69)**	0.54 (0.38–0.77)**	0.50 (0.39–0.64)**	0.42 (0.34–0.51)**	0.54 (0.39–0.74)**	1.13 (0.92–1.40)	0.50 (0.41–0.62)**	1.24 (0.82–1.89)	0.58 (0.54–0.61)**
OBC	1.07 (0.83–1.37)	0.79 (0.71–0.89)**	0.55 (0.39–0.79)**	0.80 (0.64–1.00)	1.11 (0.92–1.34)	0.86 (0.62–1.19)	1.16 (0.97–1.39)	0.80 (0.74–0.87)**	1.04 (0.84–1.28)	0.99 (0.94–1.04)
Other (Ref.)										
**Religion**										
Hindu (Ref.)										
Muslim	0.23 (0.18–0.29)**	0.49 (0.44–0.54)**	1.69 (0.96–2.95)	0.67 (0.58–0.77)**	0.89 (0.71–1.11)	0.45 (0.24–0.84)*	0.61 (0.52–0.73)**	0.68 (0.63–0.73)**	0.75 (0.58–0.97)*	0.57 (0.55–0.60)**
Others	0.66 (0.50–0.87)**	0.54 (0.19–1.57)	1.26 (0.83–1.93)	0.91 (0.78–1.07)	1.26 (0.57–2.81)	0.52 (0.41–0.65)**	2.37 (1.04–5.44)*	1.72 (0.81–3.66)	0.80 (0.39–1.65)	0.86 (0.78–0.95)**
**Age (years)**										
15–24 (Ref.)										
25–34	0.64 (0.55–0.76)**	0.75 (0.69–0.81)**	0.78 (0.69–0.89)**	0.75 (0.68–0.84)**	0.81 (0.73–0.89)**	0.68 (0.58–0.80)**	0.71 (0.62–0.80)**	0.81 (0.76–0.86)**	0.74 (0.62–0.87)**	0.79 (0.76–0.81)**
35–49	0.42 (0.34–0.53)**	0.55 (0.49–0.61)**	0.64 (0.51–0.79)**	0.63 (0.53–0.75)**	0.62 (0.53–0.73)**	0.54 (0.43–0.68)**	0.52 (0.43–0.62)**	0.66 (0.60–0.72)**	0.58 (0.43–0.78)**	0.63 (0.59–0.66)**
**Age at marriage (years)**										
Below 18	0.68 (0.59–0.80)**	0.84 (0.78–0.91)**	0.66 (0.59–0.75)**	0.78 (0.71–0.87)**	0.75 (0.69–0.82)**	0.64 (0.56–0.74)**	0.73 (0.65–0.81)**	0.82 (0.78–0.87)**	0.78 (0.66–0.92)**	0.78 (0.76–0.80)**
18 and above (Ref.)										
**Education**										
No education (Ref.)										
Primary	1.17 (0.95–1.43)	1.21 (1.08–1.35)**	1.13 (0.96–1.32)	1.33 (1.15–1.54)**	1.24 (1.10–1.39)**	1.75 (1.44–2.12)**	1.34 (1.15–1.57)**	1.18 (1.09–1.27)**	1.28 (0.99–1.64)	1.24 (1.18–1.29)**
Secondary	2.34 (1.96–2.81)**	1.71 (1.54–1.90)**	1.46 (1.24–1.71)**	2.07 (1.82–2.34)**	1.57 (1.40–1.77)**	2.84 (2.36–3.42)**	2.03 (1.71–2.40)**	1.73 (1.61–1.86)**	1.70 (1.36–2.12)**	1.83 (1.75–1.90)**
Higher	5.17 (2.59–10.31)**	3.29 (2.45–4.42)**	4.94 (3.05–8.00)**	4.08 (2.88–5.77)**	3.21 (2.09–4.94)**	3.19 (1.70–6.02)**	4.33 (2.87–6.53)**	3.70 (3.21–4.27)**	3.72 (2.62–5.28)**	3.77 (3.42–4.16)**
**Wealth index**										
Poorest (Ref.)										
Poorer	1.78 (1.51–2.09)**	1.49 (1.36–1.63)**	1.56 (1.33–1.83)**	1.44 (1.26–1.64)**	1.71 (1.53–1.91)**	1.50 (1.26–1.78)**	1.34 (1.15–1.56)**	1.21 (1.12–1.30)**	1.19 (0.86–1.64)	1.45 (1.39–1.51)**
Middle	2.57 (2.02–3.28)**	1.85 (1.62–2.13)**	1.68 (1.38–2.05)**	1.52 (1.28–1.82)**	2.40 (2.03–2.82)**	3.13 (2.35–4.17)**	1.51 (1.26–1.82)**	1.25 (1.15–1.37)**	1.47 (1.05–2.05)**	1.69 (1.60–1.78)**
Richer	3.94 (2.66–5.83)**	2.30 (1.88–2.82)**	1.92 (1.50–2.45)**	2.68 (2.04–3.53)**	2.69 (2.18–3.31)**	3.93 (2.46–6.29)**	1.77 (1.42–2.21)**	1.55 (1.39–1.73)**	2.11 (1.47–3.02)**	2.07 (1.94–2.21)**
Richest	13.61 (4.16–44.48)**	3.14 (2.09–4.72)**	3.35 (2.38–4.70)**	4.49 (2.88–7.01)**	6.37 (4.48–9.06)**	2.96 (1.55–5.65)**	3.12 (2.29–4.24)**	2.50 (2.18–2.86)**	5.18 (3.37–7.95)**	3.45 (3.15–3.78)**
**Mass media exposure**										
No (Ref.)										
Partial	1.15 (0.98–1.35)	1.02 (0.93–1.11)	1.19 (1.02–1.40)*	1.14 (1.01–1.28)*	1.32 (1.19–1.46)**	1.27 (1.09–1.48)**	1.28 (1.11–1.46)**	1.05 (0.98–1.12)	1.11 (0.88–1.41)	1.15 (1.11–1.19)**
Full	0.92 (0.63–1.36)	1.08 (0.89–1.30)	1.16 (0.81–1.64)	1.29 (0.94–1.77)	1.51 (1.16–1.96)**	2.18 (1.04–4.56)*	1.51 (0.96–2.37)	1.24 (1.04–1.48)*	1.30 (0.87–1.95)	1.31 (1.19–1.44)**
***Intermediate factors***										
**Pregnancy complication**										
No (Ref.)										
Yes	1.01 (0.87–1.18)	1.09 (1.02–1.17)*	1.36 (1.21–1.53)**	1.04 (0.94–1.15)	1.10 (1.01–1.20)*	1.33 (1.16–1.52)**	1.28 (1.14–1.44)**	1.09 (1.03–1.15)**	1.34 (1.15–1.56)**	1.13 (1.10–1.17)**
**Met with CHW**										
No (Ref.)										
Yes	1.81 1.57–2.08)**	1.65 (1.53–1.78)**	1.41 (1.23–1.61)**	1.59 (1.44–1.75)**	1.65 (1.51–1.80)**	1.45 (1.23–1.70)**	1.80 (1.60–2.03)**	1.59 (1.51–1.68)**	1.50 (1.29–1.74)**	1.63 (1.58–1.68)**
**Distance to health facility**										
No problem (Ref.)										
Small problem	0.87 (0.71–1.07)	1.03 (0.93–1.15)	1.07 (0.92–1.24)	0.96 (0.82–1.13)	0.89 (0.79–1.01)	0.91 (0.73–1.13)	1.01 (0.88–1.17)	0.96 (0.90–1.03)	1.19 (0.97–1.46)	1.00 (0.95–1.04)
Big problem	0.56 (0.46–0.69)**	0.70 (0.63–0.77)**	0.77 (0.66–0.89)**	0.77 (0.66–0.91)**	0.89 (0.79–1.00)	0.62 (0.51–0.77)**	0.83 (0.72–0.96)*	0.88 (0.82–0.94)**	0.81 (0.67–0.98)*	0.79 (0.76–0.82)**
***States***										
Assam										2.47 (2.24–2.73)**
Bihar										2.42 (2.22–2.63)**
Chhattisgarh										1.78 (1.62–1.97)**
Jharkhand										1.81 (1.65–1.98)**
Madhya Pradesh										3.50 (3.21–3.82)**
Odisha										4.62 (4.18–5.11)**
Rajasthan										4.87 (4.43–5.34)**
Uttar Pradesh										1.78 (1.65–1.93)**
Uttarakhad (Ref.)										

Significance level: **p < 0.01; *p < 0.05.

Abbreviations: Ref: Reference category; OR: Odds ratio; CI: Confidence interval

### Accessibility and barriers to delivery in a health facility

Table 4 illustrates different limitations/barriers to delivery at health facilities (nine main reasons for not delivering in a health facility) across nine LPS. Overall, 38% of women were not delivering at health facilities as they felt it was not necessary and this proportion was highest in Chhattisgarh (47%) followed by Jharkhand (43%) and Rajasthan (42%). About 19% of women could not deliver at an institution due to family constraints and this proportion was found to be highest in Bihar (25%) followed by Uttar Pradesh (18%), Assam (18%), Madhya Pradesh (17%) and Rajasthan (17%). Additionally, about 17% of women expressed distance or lack of transportation and costs (16%) to be challenging in accessing health facilities for the delivery along with other reasons like facility closures (10%), poor service/trust issues (6%), and others. Closely looking at the observations, it is evident that the proportions for each of the reasons vary across nine LPS of India (state-specific reasons), where ‘not delivering due to too far or no transportation’ was highest in Madhya Pradesh (31%) and reason like ’costs too much’ was highest in Assam (24%) among several others.

**Table 4.** Percentage distribution of reasons for not delivering in a health facility across LPS, NFHS-4 2015–16

Reasons for not delivering in a health facility	Assam	Bihar	Chhattisgarh	Jharkhand	Madhya Pradesh	Odisha	Rajasthan	Uttar Pradesh	Uttarakhad	Overall
Costs too much	23.9	15.5	7.5	17.5	12.8	22.7	9.4	15.9	15.8	15.5
Facility not open	12.1	11.4	6.7	8.2	12.0	14.3	9.1	8.4	13.2	10.0
Too far/no transportation	25.5	15.3	27.5	24.4	30.5	24.2	20.2	10.8	15.8	17.3
Don’t trust facility/poor service	3.1	6.5	4.0	5.7	5.6	4.5	3.8	7.4	8.7	6.2
No female provider	2.3	4.5	1.9	3.3	3.7	2.2	4.3	3.1	4.1	3.5
Husband/Family did not allow	17.7	24.7	16.0	14.9	16.6	9.3	16.7	18.0	12.3	18.9
Not necessary	36.5	37.6	47.4	43.0	24.5	34.0	42.0	39.6	39.8	38.2
Not customary	8.5	3.6	4.6	4.1	2.9	3.3	4.2	4.2	4.5	4.1
Other	0.9	6.1	4.8	7.9	12.0	3.6	10.0	14.1	8.1	9.4

Table 5 shows the extent to which distance to a health facility was a challenge for women in accessing medical care across nine LPS. It is observed that distance was a major barrier to seek treatment or medical care from a health facility in all nine LPS. Overall, 38% of women felt that distance was a ‘big problem’ in seeking medical care. Nearly half of the women from Jharkhand reported that distance was a ‘big problem’ (49%) followed by Bihar (44%) and Odisha (40%), indicating that a majority of women faced difficulties in accessing medical care in these states, leading to low maternity care utilization including institutional delivery service. On the other hand, the highest share of women residing in Uttarakhand indicated that distance was ‘not a problem’ (38%) followed by Rajasthan (35%) and Chhattisgarh (33%).

**Table 5.** Perceived distance to the health facility across LPS, NFHS-4 2015–16

States	No problem	Small problem	Big problem
Assam	23.2	42.7	34.1
Bihar	18.9	36.7	44.4
Chhattisgarh	33.4	34.5	32.1
Jharkhand	17.1	34.0	48.9
Madhya Pradesh	32.3	31.3	36.4
Odisha	21.7	38.0	40.2
Rajasthan	34.8	35.3	29.8
Uttar Pradesh	31.4	32.4	36.2
Uttarakhad	38.2	28.9	32.9
Overall	27.5	34.6	37.9

Table 6 shows the interaction model assessing the combined influence of place of residence and distance to the health facility on institutional delivery. It is found that after controlling for socio-demographic characteristics of women and pregnancy-related factors, women from urban areas who felt accessing health facilities was a big problem had lower odds of utilizing institutional delivery (AOR: 0.89; 95% CI: 0.81, 0.99). The odds of delivering in an institution were further lowered for women living in rural areas (AOR: 0.75; 95% CI: 0.70, 0.81).

**Table 6.** Interaction models (bivariate and multivariate) assessment to determine the impact of place of residence and distance to the health facility on institutional delivery utilization in LPS, NFHS-4 2015–16

Place of residence × Distance to the health facility	Crude OR (95% CI)	Adjusted OR (95% CI)
Urban × no problem (Ref.)		
Urban × a small problem	0.80 (0.74–0.87)**	1.03 (0.95–1.13)
Urban × a big problem	0.56 (0.51–0.62)**	0.89 (0.81–0.99)*
Rural × no problem	0.59 (0.55–0.63)**	1.00 (0.93–1.07)
Rural × a small problem	0.46 (0.43–0.49)**	0.98 (0.92–1.05)
Rural × a big problem	0.30 (0.29–0.32)**	0.75 (0.70–0.81)**

Significance level: **p < 0.01; *p < 0.05.

Note: Adjusted model was controlled for caste, religion, age, age at marriage, education, wealth status, exposure to mass media, pregnancy complication, and met with CHW.

Abbreviations: OR: Odds ratio; CI: Confidence interval

### JSY coverage across LPS and by socio-demographic characteristics

**Figure 7.**
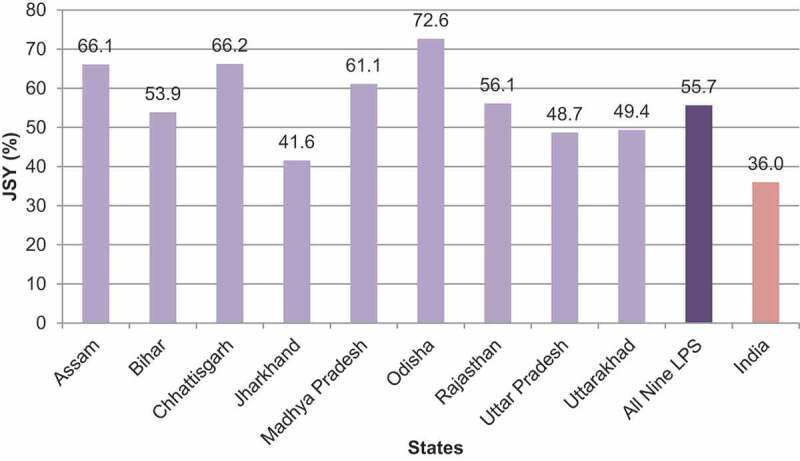
Coverage of JSY scheme (%) across LPS, NFHS-4 2015–16

Although India has launched the JSY scheme as part of the safe motherhood programme, results show that the coverage of the scheme is not the same across all LPS and the utilization of the scheme is unequal and uneven by the socio-demographic profile of the study population. Figure 7 indicates that states like Odisha which had a high institutional delivery coverage utilized the JSY scheme the most (institutional delivery: 87%; JSY: 73%) followed by Assam (institutional delivery: 73%; JSY: 66%) and Chhattisgarh (institutional delivery: 73%; JSY: 66%). On the other hand, Jharkhand had the lowest JSY coverage where institutional delivery was also low (institutional delivery: 64%; JSY: 41%). Table 7 shows a statistically significant association between all socio-demographic characteristics of the study population and the utilization of the JSY scheme. The utilization of JSY services was substantially higher among rural residents (60%), socially marginalized caste groups (SC: 62%; ST: 66%), and those who were Hindu (57%). The coverage of JSY was more among child-married women as compared to their adult-married counterparts (61% vs. 52%). It is also observed that women who had no formal education (62%), belonged to lower wealth quintiles (poorest: 66%; poorer: 64%), and those who had no exposure to mass media (63%) were more likely to get assistance from the JSY scheme. The multivariate logistic regression analysis also revealed similar results as found in the bivariate analysis. The utilization of assistance from the JSY scheme was greater among socio-economically marginalized sections since the scheme was initiated targeting disadvantaged groups of the population to improve institutional delivery coverage among them.

**Table 7.** The percentage distribution (%) and multivariate logistic analysis assessing the likelihood of JSY scheme utilization by socio-demographic characteristics of women in LPS, NFHS-4 2015–16

Characteristics	Total N	JSY (%)	OR (95% CI)
**Place of residence†**			
Urban	19,137	39.3	1.00
Rural	64,511	60.2	1.32 (1.26–1.37)**
**Caste†**			
SC	15,757	62.4	1.31 (1.25–1.38)**
ST	11,172	66.4	1.51 (1.43–1.60)**
OBC	39,612	55.1	1.14 (1.09–1.19)**
Other	14,915	43.1	1.00
**Religion†**			
Hindu	70,928	56.9	1.00
Muslim	10,897	48.3	0.75 (0.72–0.79)**
Other	1823	49.7	0.77 (0.69–0.85)**
**Age†**			
15–24	30,092	54.3	1.00
25–34	46,010	56.3	1.24 (1.20–1.28)**
35–49	7546	57.1	1.19 (1.13–1.26)**
**Age at marriage†**			
Below 18	33,837	60.8	1.07 (1.04–1.10)**
18 and above	48,863	51.8	1.00
**Education†**			
No education	25,584	62.4	1.64 (1.54–1.75)**
Primary	12,200	61.6	1.73 (1.62–1.84)**
Secondary	36,779	54.6	1.65 (1.56–1.74)**
Higher	9085	32.0	1.00
**Wealth index†**			
Poorest	23,711	65.5	3.41 (3.19–3.65)**
Poorer	20,822	63.6	3.22 (3.03–3.42)**
Middle	15,464	56.6	2.58 (2.43–2.74)**
Richer	12,386	46.0	1.87 (1.76–1.98)**
Richest	11,265	27.7	1.00
**Mass media exposure†**			
No	24,392	62.6	1.00
Partial	53,988	53.4	1.19 (1.14–1.24)**
Full	5268	44.0	1.08 (1.00–1.16)*

Note: †Differences were significant at *p* < 0.01 (derived from Pearson’s chi-square test).

Significance level for multivariate analysis: **p < 0.01; *p < 0.05.

Abbreviations: OR: Odds ratio; CI: Confidence interval

## Discussion

Despite several ‘Safe Motherhood’ programmes initiated by the government, the MMR remains higher in LPS than the nation’s average. Delivery at a health institution is a key intervention to avert the risk of maternal mortality due to childbirth-related complications. While states like Madhya Pradesh, Odisha, and Rajasthan are relatively in a better position in institutional delivery coverage, states like Chhattisgarh, Jharkhand, Bihar, Uttar Pradesh, and Uttarakhand are still lagging. Although Jharkhand stands out as the worst-performing state in institutional delivery as well as the JSY scheme coverage among all LPS, the state has managed to reduce the MMR to 71 deaths per 100,000 live births in 2016–18. This contradictory finding further reinforces to explore whether the improvement in institutional delivery and JSY utilization is effective to reduce maternal mortality. Although some previous studies have reported no significant relationship between the rise in institutional delivery and the reduction of maternal mortality in LPS [26,47], nevertheless in-facility deliveries should be increased as it effectively manages any emerging unpredictable delivery complications in an enabling environment equipped with adequate SBA and optimal emergency obstetric care functionality [19,20]. However, we have not intended to reaffirm this association; rather our study provides insights about important determinants of and barriers to institutional delivery in the context of the JSY scheme in nine focused states of India.

Our findings are similar to Barman et al. [12] where they also found that women aged 15–24 years tend to deliver more at any health facility than women aged 25–34 and 35–49 years, which could be because younger women utilize more antenatal care services (more exposed to knowledge about safe delivery services through antenatal programme counselling) than older women and the level of education could be an associated factor too. With regard to age at marriage, child married women (married before 18 years) were found to be less likely to deliver at any health facility compared to women who got married at 18 years or later. The results corroborated with a previous study of India by Paul & Chouhan [48]. This is probably because the completion of basic education could be comparatively higher among women who got married at or after legal age where they were more exposed to the knowledge and practices about safe maternity care. Studies also suggest that younger brides often face restrictive behaviour and marital violence by the husband and in-laws, leading to low self-efficacy and a lack of decision-making autonomy that could adversely impact receiving maternity care [49–51]. Similarly, our findings are parallel with other studies which also highlighted that women with higher education backgrounds were more likely to utilize institutional delivery than women with no or relatively less educational background [29,52–54]. The reason being the same as exposure to higher-level education potentially enables women to be aware of the clinical health benefits of delivering at a health facility. Socio-cultural factors such as religion and caste emerged as significant predictors of institutional delivery services where women belonged to Muslim and SC/ST did not prefer to deliver at an institution. Barman et al. [12], Paul & Pandey [53], and Paul & Chouhan [54] also found that compared to Hindus, Muslim women were less likely to use institutional delivery services. Owing to their poor socio-economic condition, women from socially disadvantaged sections experience multiple forms of difficulties regarding access to proper maternity care. Mass media exposure enables women to be more aware of safe maternity care benefits. It enhances proper knowledge about sexual reproductive health and leads to positive healthcare-seeking behaviour. Our findings are in tune with other studies that women who are exposed to mass media have a higher likelihood of using institutional delivery services than women with no exposure at all [12,54]. Concerning intermediate factors, meeting with a CHW during pregnancy is a strong predictor in choosing to deliver at a health facility and our findings corroborate with other studies which highlighted CHWs like ASHA as the ‘agent of change’ and indicated that women who had exposure to ASHAs were more likely to utilize safe delivery services than women who did not meet any CHW like ASHA [28,53].

Since the JSY scheme was mainly developed targeting socioeconomically disadvantaged women to improve institutional delivery coverage, women from the lower wealth quintiles (poor economic backgrounds) and rural residences were more likely to utilize the JSY cash incentive programme. Despite the recorded achievements of the JSY scheme to bring women into an institution for delivery care, women from low socioeconomic backgrounds were still less likely to utilize the services. While studies have found that JSY cash incentive is effective and encouraging for delivering in a health facility, improved targeted attention to the quality of care is needed for much better results [14,55]. Sidney et al. [28] found that although more than half of the women found the cash incentive scheme effective, few women found it is not motivating enough and rather thought obtaining the cash benefit would be challenging. A recent study conducted in India indicates that the use of maternity care (i.e. contraceptive use, breastfeeding practices, and postnatal care) is significantly higher among JSY-beneficiaries than non-beneficiaries, even after controlling for various socio-demographic characteristics [56]. Since the goal of the JSY scheme is to reduce maternal mortality, several previous studies have evaluated the impact of this scheme on reducing maternal deaths [14,57]. Ng et al. [47] in their study in Madhya Pradesh and Randive et al. [26] using Annual Health Survey data in nine LPS found that JSY-supported institutional deliveries are non-effective to reduce maternal mortality.

Some of the state-specific initiatives like MAMATA, a conditional maternity benefit scheme by the Government of Odisha, *Indira Gandhi Matritva Sahyog Yojana* by the Ministry of Women and Child Development for pregnant and lactating women implemented in the state of Bihar, and *Matra Evam Shishu Swasthya Sanrakshan Abhiyan* in 2015 by the Government of Uttar Pradesh were introduced to increase institutional delivery coverage in these states [58–60]. An effort of the Government of Madhya Pradesh entitled ‘Gram Arogya Kendra’ to provide health service at the community level also promotes institutional delivery services [61].

In the present study, several barriers such as long-distance/unavailability of transport facilities, high costs, restricted mobility, and perceiving institutional delivery as unnecessary have been reported as the main reasons for not delivering at a health institution. Apart from our mentioned reasons, there could be diverse reasons/factors influencing not to deliver at a health facility. It is imperative to contextualize the barriers to delivery in a health facility in light of the ‘three delays model’ introduced by Thaddeus and Maine in 1994 [62]. The first phase of the model which is delays in deciding to seek care (e.g. high costs, poor quality of service, family restriction, and perceiving as not necessary and customary) is the most common reason for not delivering in a health facility in the study area. The second phase of delay (delay in reaching the health facility) could be high due to long distances or a lack of available (emergency) transportation. Delay in providing care inside the health facility could also contribute to lower rates of institutional delivery since a significant proportion of women reported poor service and a lack of female providers inside health care facilities. A previous study of India also identified the first type of delay (delay in seeking care) as the major contributor to increased maternal mortality [63]. Another study in rural areas of Haryana, India found that household and transport-related delays (first and second phases of delays) are the major contributors to avertable neonatal deaths [64]. In Egypt, the third phase of delay is the most significant reason for maternal deaths in a tertiary hospital [65]. The findings of our study also indicate that place of residence and distance to the health facility (Table 6) had a statistically significant interactive association with institutional delivery in which delivering at any health institution is challenging when the distance to a health facility is considered as a ‘big problem’ irrespective of the place of the residence (urban/rural). Similarly, Kumar et al. [64] also found that long-distance deters uptake of institutional delivery services where each extra one kilometre (between the place of residence and reachable/accessible health facility) reduces the chances of institutional delivery by 4.4%. Their study findings also highlight that persistent perpetuated individual and structural level barriers like financial insecurity (e.g. high OOPs), poor quality of care, lack of transportation, and its associated costs along with underpinned socio-cultural norms continue to hinder the utilization of institutional delivery services [66].

### Limitations and strengths

The results of the present study should be interpreted cautiously along with its limitations. We adopted a cross-sectional study design; therefore, causality cannot be assumed between predictors and outcome of interest (institutional delivery) in this study. The dataset utilized in this study did not cover all the indicators such as type and distribution of available health facilities and presence of healthcare professionals at all healthcare tiers (primary, secondary, and tertiary) which might be important determinants in the decision making regarding institutional delivery. Data regarding distance to health facilities and pregnancy complications under intermediate factors were also limited. We used perceived distance to the health facility rather than actual distance in the analysis as a proxy to understand the question of accessibility of utilizing institutional delivery services due to the lack of direct information in the dataset. Along with this, not all the variables that determine institutional delivery among women were reviewed in our present study owing to the objectives and scope of the study. Also, the performance of the JSY and all other results shown in the study are based on the evidence preceding five years when the data was collected. Hence, there could be significant advancements of JSY and changes in other included variables directly affecting uptake of safe delivery services which were not captured in the present study. Other national-level initiatives like *Pradhan Mantri Matru Vandana Yojana* and *Ayushmann Bharat* may also have a direct or indirect influence on the utilization of institutional delivery services which were not included in the present study as these programmes were launched post-NFHS-4, 2015–16 survey completion. Additionally, NFHS is a retrospective study design, and it is likely that the data reported in the NFHS might suffer from non-systematic reporting bias and systematic recall bias. Apart from the limitations, our study contributes substantially to the literature which has significant value for policy framework. We made a comprehensive analysis on institutional delivery in nine LPS using an adequate number of samples that are representative of each state. Therefore, results derived from the analysis are robust and consistent. Moreover, our study tries to explore the question of accessibility from the perspectives of distance to the health facility that provides a unique opportunity to make effective interventions to improve these services in the study setting. Further in-depth qualitative study is needed to understand the persistent low coverage of institutional deliveries across LPS of India.

## Conclusion

With the coverage of institutional deliveries being persistently low in LPS, an integrated and targeted state-specific intervention should not only focus on increasing the number of public health facilities but also improving its associated quality of care (person-centred maternity care). Although India has recently institutionalized midwifery care into the health system to strengthen the quality of maternal and new-born services in the birthing centres, inadequate clinical training and insufficient skilled human resources restrained the quality of available maternity services resulting in low coverage of institutional deliveries. Awareness about benefits of the JSY scheme (JSY replicated state-specific schemes), process of incentive disbursement (accountability and transparency), and (emergency) transportation availabilities should be strengthened as they have been identified as major barriers to access institutional deliveries under JSY. Efficient and increased investment in the public health system (adequate provision of training, recognition, and consistent retention of skilled personnel) at all tiers (specifically strengthening primary level PHCs and emergency referral networks which are most accessible and affordable among the disadvantaged groups) is imperative to effectively reduce financial inequities of service use and ensuring optimal care for mothers and new-borns. Since deep-rooted socio-cultural norms influence patterns of safe delivery utilization and JSY has not been proven sufficient to close the gap between low and high-performing states, targeted and integrated socio-behavioural change interventions are crucial for improving institutional delivery coverage.

## Paper context

Despite the implementation of several national-level interventions, institutional delivery coverage remains unsatisfactory in low performing states (LPS) of India leading to a high burden of maternal mortality. Apart from socio-demographic characteristics, meeting with community health workers (CHWs) during pregnancy and distance to the health facility emerged as important predictors of institutional delivery service. Significant geographical variations and disparities across socio-economic groups regarding utilization of institutional delivery and JSY services suggest further strengthening of state-specific policies and programmes.
